# Inclusion Complex of a Cationic Mono-Choline-β-Cyclodextrin Derivative with Resveratrol: Preparation, Characterization, and Wound-Healing Activity

**DOI:** 10.3390/ijms26146911

**Published:** 2025-07-18

**Authors:** Sonia Pedotti, Loredana Ferreri, Giuseppe Granata, Giovanni Gambera, Nicola D’Antona, Claudia Giovanna Leotta, Giovanni Mario Pitari, Grazia Maria Letizia Consoli

**Affiliations:** 1 CNR-Institute of Biomolecular Chemistry, 95126 Catania, Italy; sonia.pedotti@cnr.it (S.P.); loredana.ferreri@cnr.it (L.F.); giovanni.gambera@cnr.it (G.G.); nicola.dantona@cnr.it (N.D.); 2 Dream Factory Lab, Vera Salus Ricerca S.r.l., 96100 Siracusa, Italy; claudia.leotta@verasalusricerca.it (C.G.L.); giovanni.pitari@verasalusricerca.it (G.M.P.); 3 J4Med Lab, 95126 Catania, Italy

**Keywords:** cyclodextrin, inclusion complex, molecular modeling, resveratrol, wound-healing

## Abstract

Resveratrol is one of the most extensively studied natural products due to its pleiotropic health benefits. However, its low water solubility and limited stability hinder its application in the nutraceutical, cosmetic, and pharmaceutical sectors. In this work, we investigated the ability of a cationic mono-choline-β-cyclodextrin derivative to complex *trans*-resveratrol. The complex was prepared using a phase solubility method without using organic solvents and was found to be stable after freeze-drying. The complex was characterized by a phase solubility study, NMR spectroscopy, and molecular modeling simulations, which revealed a 1:1 stoichiometry, a stability constant of 2051 M^−1^ (*K_C_*), and structural details. Complexation improved resveratrol’s solubility and dissolution rate, reduced its photoinduced *trans*-to-*cis* isomerization, and preserved its radical scavenging activity. The wound-healing activity of the complex was demonstrated via in vitro experiments on human keratinocyte cells.

## 1. Introduction

Resveratrol (3,5,4′-trihydroxy-*trans*-stilbene; RES) is a natural secondary metabolite from the stilbenoid family that has attracted significant scientific attention due to its wide-ranging health-promoting effects [1]. RES has shown multiple biological activities, including anti-inflammatory [2], antioxidant [3], antibacterial and antifungal [4], anti-aging [5], anticancer [6], cardioprotective [7], and neuroprotective [8] properties. Additionally, RES and its derivatives have demonstrated excellent cosmetic potential, particularly as whitening agents for treating melanin-related skin spots [9]. Although the precise molecular mechanisms of RES’s biological activities are not well understood, it is well established that RES acts as a potent free radical scavenger. The biologically active form of RES is the *trans*-isomer, which in nature is found in grapes, berries, peanuts, pineapples, quinoa, and other plants [10]. RES is an off-white powder soluble in dimethyl sulfoxide and ethanol but practically insoluble in water (∼0.03 mg/mL at 25 °C) [11], as defined by the European Pharmacopeia. Poor water solubility, lipophilic nature, and sensitivity to environmental factors such as pH, temperature and light, and consequent low bioavailability and handling limit the use of RES in the pharmaceutical industry. For example, the high hydrophobicity of RES precludes its formulation in aqueous systems without organic solvents. The inclusion of RES in molecular carriers reveals a promising strategy to overcome these limitations. For instance, encapsulating RES in polymeric aminoalkyl-methacrylate nanoparticles has improved its solubility and hepatoprotective effects [12].

Similarly, inclusion complexes with cyclodextrins (CDs) have shown great potential to enhance the application of RES in the nanomedicine and biotechnology field [13,14]. β-CD and methylated β-CD have also been employed to optimize the bioproduction of RES [15]. Inclusion of RES into native CDs and their derivatives, such as methylated α-CD and β-CD [16], 2,6-permethylated-β-CD, sulfobutyl ether-β-CD [17], and hydroxypropyl-β-CD (HP-β-CD) [18], has improved its aqueous solubility, chemical stability, and bioavailability without compromising its pharmacological properties [19]. The inclusion in CDs protected RES from degradation caused by oxidative stress, UV light, and heat, while enhancing cellular uptake [20]. Given its antioxidant, anti-inflammatory, and antibacterial properties, RES is also being explored for applications in wound-healing therapies [21,22,23,24,25]. Recently, Yu et al. demonstrated that β-CD can effectively deliver RES to the stratum corneum [26], improving its low dermal penetration.

Cyclodextrins are cyclic oligosaccharides composed of 1,4-linked α-D-glucopyranose units [27]. The CD family offers oligomers differing in the number of the glucose units forming the macrocycle (6, 7, and 8 units for α, β, and γ-CD, respectively) and consequently in the size of their cavities. Notably, CDs and some of their derivatives are approved by the US Food and Drug Administration as food additives and pharmaceutical excipients. CDs possess a truncated cone-shaped structure, with hydroxyl groups on the outer surface conferring hydrophilicity, and hydrogen atoms (H3, H5) within the cavity contributing to hydrophobicity.

Previously, we reported the synthesis and characterization of a cationic β-CD functionalized with a single choline group (β-CD-Chol). The positively charged choline moiety enhances the aqueous solubility of the β-CD scaffold and imparts mucoadhesive properties useful for prolonging drug residence time at application sites [28]. The presence of a complexing cavity, its non-toxicity, and the potential of the cationic choline group to interact with cellular membranes via electrostatic interactions or by binding choline transporters [29,30,31,32] could make β-CD-Chol a candidate for RES delivery. While complexation of RES with anionic sulfobutyl ether-β-CD has been explored [33], to our knowledge, no evidence exists regarding the inclusion complex of RES with cationic β-CDs.

In this study, we prepared and characterized the inclusion complex of β-CD-Chol and RES. The complex was prepared using the phase solubility method and was characterized by UV–vis spectrophotometry and NMR spectroscopy. Stoichiometry, stability constant, complexation coefficient, solubility enhancement factor, and dissolution rate were evaluated. Furthermore, 1D- and 2D-NMR spectra and molecular modeling simulations provided insights into the geometry of the complex. The effects of complexation on light-induced *trans*-to-*cis* RES isomerization, radical scavenging activity, and wound-healing activity were also assessed.

## 2. Results and Discussion

### 2.1. Synthesis of Mono-Choline-β-Cyclodextrin Derivative (β-CD-Chol)

**Figure 1:**
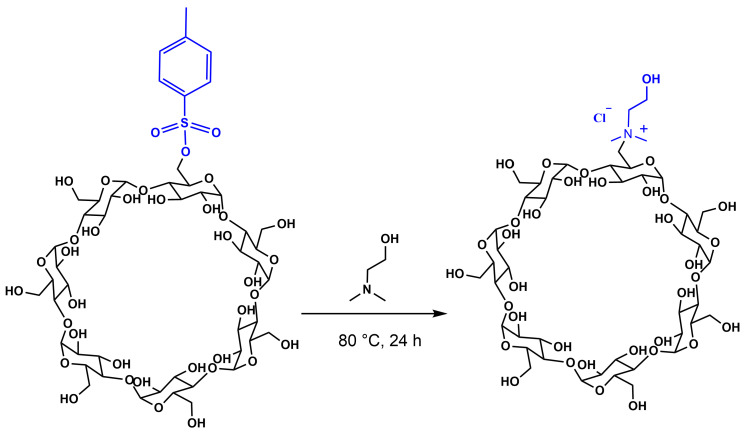
Procedure for the synthesis of the mono-choline-β-CD derivative (β-CD-Chol). The chloride salt was obtained after passage through an ion exchange resin (chloride form).

The mono-choline-β-cyclodextrin derivative (β-CD-Chol) was synthesized as previously reported [28] and schematically illustrated in Figure 1. Briefly, commercially available β-CD was mono-tosylated at one of its primary hydroxyl groups [34] and subsequently reacted with dimethylaminoethanol to yield the designed cyclodextrin derivative. The success of the monofunctionalization was confirmed by nuclear magnetic resonance (NMR) spectroscopy. The proton spectrum displayed the characteristic signals of the cyclodextrin scaffold along with additional signals corresponding to the choline substituent (N-CH_3_ and NCH_2_CH_2_OH). The integration of these signals was consistent with the mono-functionalization.

### 2.2. Preparation and Characterization of the β-CD-Chol/RES Inclusion Complex

The inclusion complex between β-CD-Chol and RES was prepared using the phase solubility method without the use of organic solvent. An excess of solid RES was added to an aqueous solution of β-CD-Chol (10 mM, phosphate-citrate buffer, pH 6). The suitability of the complex for lyophilization allowed its recovery in solid state, useful for improved handling, stability, and long-term storage.

#### 2.2.1. UV–Vis Characterization and Phase Solubility Analysis

The absorption spectrum of the complex displayed the typical band of RES at 306 nm (π→π* transition due to the conjugated double bond) and, from the absorbance value at this wavelength, the amount of RES in the complex was determined to be 100 µg/mL *per* 2 mg of β-CD-Chol.

**Figure 2:**
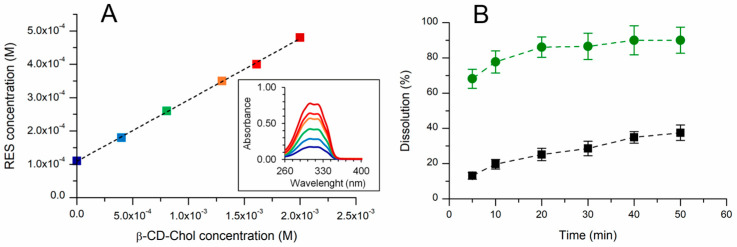
(**A**) Linear phase solubility diagram of β-CD-Chol/RES complex obtained from UV–vis spectra (16-times dilution) of RES solubilized at increasing concentrations of β-CD-Chol (inset). Different colors correspond to different concentrations. (**B**) Dissolution profile of the lyophilized β-CD-Chol/RES complex (green circles) and RES powder (black squares) in citrate-phosphate buffer (pH 6) at 37 °C (RES in the samples 0.1 mg/mL, 0.4 mM).

A phase solubility study showed that the absorption band of RES enhanced linearly with increasing β-CD-Chol concentrations (from 0 to 2.0 × 10^−3^ M). As described by Higuchi and Connors [35], RES concentration was plotted as a function of β-CD-Chol concentration. The phase solubility diagram showed an AL-type profile (Figure 2A). The linear relationship indicated the formation of a 1:1 inclusion complex, as further supported by the slope of the plot being less than unity.

From the slope of the phase solubility diagram, the stability constant of the inclusion complex (*K_C_*) was calculated to be 2051 M^−1^. This value was similar to that reported for other β-CD derivatives (2057, 1588, and 2604 M^−1^ for β-CD, HP-β-CD, and DM-β-CD, respectively) [36]. The complexation efficiency (CE) was determined to be 0.23. This CE value suggested that approximately one out of every five β-CD-Chol molecules complex RES [37].

The complexation increased the water solubility of RES from ∼0.03 mg/mL [11] to ∼4.7 mg/mL. As shown in Figure 2B, the inclusion into β-CD-Chol improved the dissolution rate of RES. The amount of RES in solution was determined to be 90% and 37.5% for the lyophilized complex and RES, respectively, after 50 min.

#### 2.2.2. NMR Characterization of the Complex and Molecular Modeling Simulations

**Figure 3:**
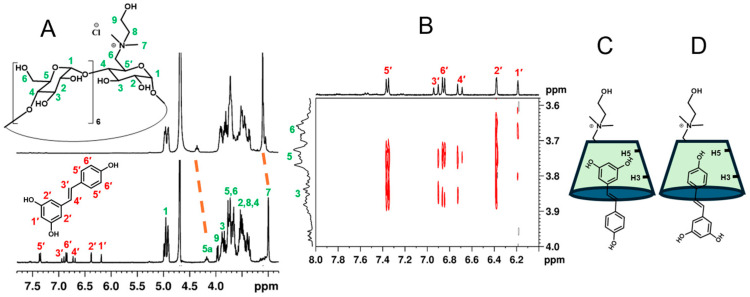
(**A**) ^1^H NMR spectra of β-CD-Chol (up) and β-CD-Chol/RES complex (bottom). (**B**) Relevant region of 2D-ROESY spectrum of β-CD-Chol/RES complex, (400.13 MHz, D_2_O, 297 K). (**C**,**D**) Schematic representations of two possible conformers for the β-CD-Chol/RES complex.

NMR is one of the most informative spectroscopic techniques for confirming formation of inclusion complexes. In β-CD, H3 and H5 protons are located inside the hydrophobic cavity; in particular, H3 is next to the wider edge and H5 is near the narrower edge. H1, H2, and H4 reside on the outer surface of the β-CD macrocycle [27]. The presence of RES signals in the proton spectrum of the complex in deuterated water (Figure 3A) was consistent with enhanced drug solubility due to the inclusion into the cyclodextrin cavity.

Upfield chemical shift changes (Δδ from 0.02 to 0.13 ppm) were observed for all cyclodextrin protons, which are coherent with a shielding effect due to RES inclusion. Multiple signals for each proton and overlaps made a precise assignment difficult. However, a clear upfield shift was detected for the N-CH_3_ protons (3.14 ppm) of the choline moiety (Δδ 0.06 ppm) and the H5a proton (4.41 ppm) of the β-CD-Chol ring bearing the choline group (Δδ 0.13 ppm). This suggests that the choline group may be involved in the complexation, or that a conformational change occurs during RES complexation.

To further investigate the structure of the complex, 2D-ROESY NMR spectra were acquired (Figure 3B). Cross-peaks between the H3 and H5 of β-CD-Chol and the alkene protons (H3′ and H4′) of RES corroborated the formation of an inclusion complex. The analysis of the cross-peaks also showed a correlation between the protons of the β-CD-Chol cavity (H3, H5) and the protons of both the aromatic rings of RES. Therefore, it is plausible that more than one geometry may be present for the inclusion complex. The correlations of H3, H5, and H6 protons of β-CD-Chol with H1′ and H2′ protons of RES indicated a geometry where the aromatic ring with two OH groups is in the cavity, oriented toward the narrow edge of the cyclodextrin (Di-OH form) (Figure 3C). The correlations of H3 and H5 protons of β-CD-Chol with H5′ and H6′ protons of RES were instead consistent with the aromatic ring with one OH group in the cavity, oriented toward the narrow edge of the cyclodextrin (Mono-OH form) (Figure 3D). The co-existence of these orientations has been reported for the inclusion complexes of RES and native β-CD [37,38,39].

**Figure 4:**
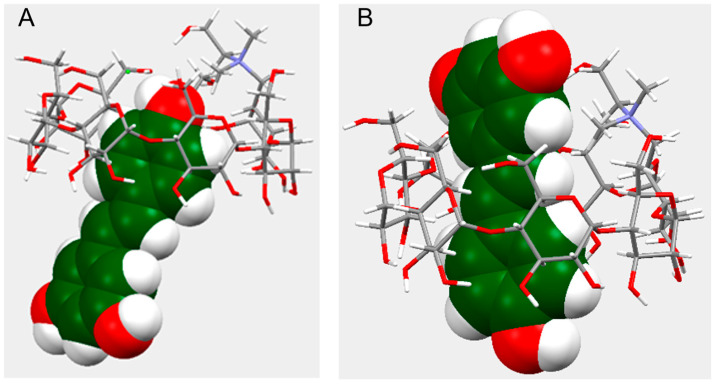
Molecular models of two possible low-energy conformers (**A**,**B**) of the β-CD-Chol/RES inclusion complex (side view).

To gain further insight on the structure of the complex, molecular modeling simulations were carried out. The lowest-energy conformations for the inclusion complex are shown in Figure 4.

Figure 4A illustrates the conformer in M-form geometry where only the phenol ring of RES is inserted into the cyclodextrin cavity (E = −337.7093 Eh). This structure agreed with the cross-peaks observed between H3 and H5 and H5′ and H6′ protons. The Di-OH form geometry, in which the ring of RES with two hydroxyl groups is towards the cyclodextrin narrow edge, instead provided the structure depicted in Figure 4B (E = −337.7067 Eh). In this conformer, the RES molecule crosses the cyclodextrin cavity. This structure supported the cross-peaks observed between the cyclodextrin protons (H3, H5, H6) and the alkene (H3′, H4′) and aromatic protons of RES. Such a structure could promote photostability due to reduced environmental exposure of the central double bond, while preserving antioxidant activity, as the hydroxyl group responsible for radical scavenging is accessible to radicals [39].

#### 2.2.3. Radical Scavenging Activity and Photostability of RES in the Complex

The radical scavenging activity of RES in the complex was assessed using the DPPH (2,2-diphenyl-1-picrylhydrazyl) assay [40,41].

**Figure 5:**
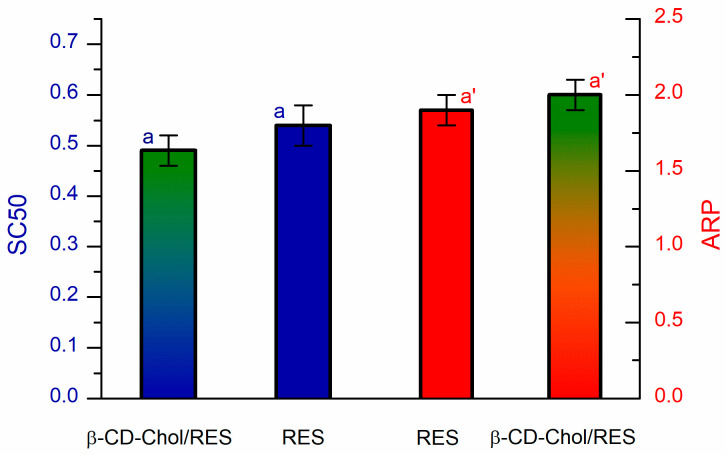
DPPH radical scavenging activity of β-CD-Chol/RES complex and RES. SC_50_ (left, blue) and ARP (right, red). The bars with gradient color refer to the complex. Values with the same superscripts are not significantly different at 0.05 level.

In Figure 5, the DPPH radical scavenging activity is reported as SC_50_ and antiradical power (ARP) values. The amount of RES required to scavenge half of the DPPH radical (SC_50_) was determined to be 0.54 ± 0.04 and 0.49 ± 0.03 (µmol of RES/µmol of DPPH) for RES and β-CD-Chol/RES complex, respectively. The not significant difference in SC_50_ values indicated that the inclusion process did not impair the antioxidant properties of RES.

Several studies have demonstrated that the *trans*-isomer of RES is more stable and resistant to degradation than the *cis*-isomer, likely due to its non-planar conformation. Both *cis* and *trans*-isomers of RES exhibit biological activity, but the *trans*-isomer has shown superior efficacy in numerous therapeutic applications [42,43,44,45,46]. Thus, preserving RES in *trans*-configuration is essential for biological applications. To evaluate the photoprotective effect of β-CD-Chol, a preliminary study was conducted to assess the photoinduced *trans*-to-*cis* isomerization of RES. Solutions of RES and β-CD-Chol/RES were exposed to UV light (λ 365 nm) or natural sunlight. The concentrations of *trans*- and *cis*-RES before and after irradiation were quantified by HPLC. The amounts of retained *trans*-RES after exposition to UV light (30 min) or sunlight (7 days) were determined to be 20 and 50% for β-CD-Chol/RES and 5 and 10% for RES, respectively. These results, which demonstrated a photoprotective effect of β-CD-Chol on RES photoinduced isomerization, would be consistent with a complex structure in which the alkene group of RES is protected within the cyclodextrin cavity [39].

### 2.3. Wound-Healing Activity

Due to its antioxidant, anti-inflammatory, and antibacterial properties, RES has been explored for wound-healing treatment. It has been demonstrated that RES can (i) accelerate wound healing by mitigating oxidative stress-induced impairment of cell proliferation and migration [23]; (ii) upregulate the expression of vascular endothelial growth factor, thereby promoting angiogenesis and endothelial cell proliferation and migration [24]; and (iii) suppress inflammation by key signaling pathways, including nuclear factor kappa B (NF-kB) and mitogen-activated protein kinase (MAPK), which are critical in the wound-healing process [25].

**Figure 6:**
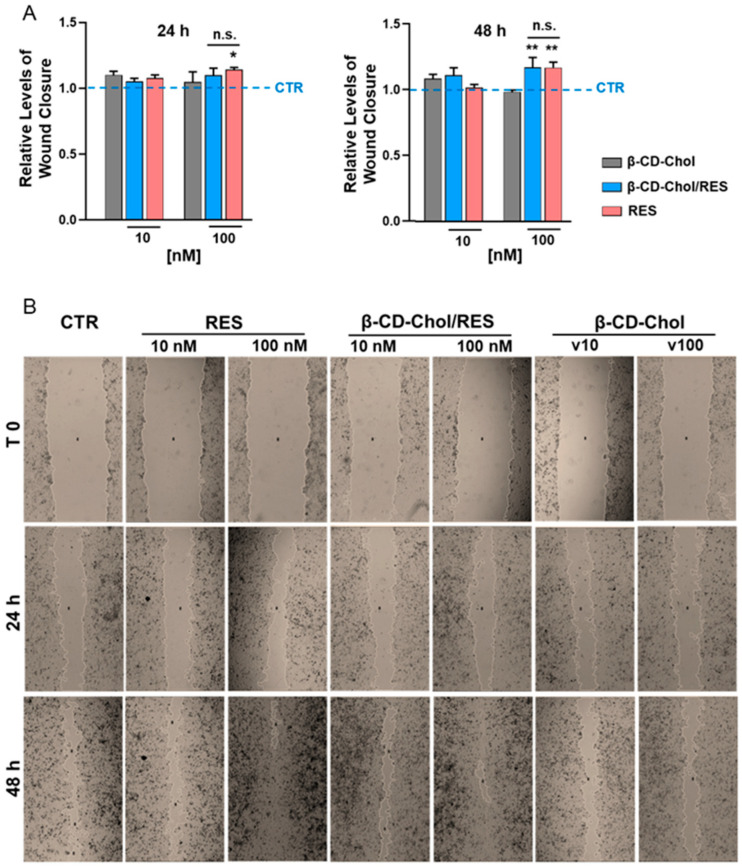
Wound-healing assays in human keratinocyte HaCaT cells. Wound closure effects after scratching and treatments with RES (10 and 100 nM) alone and in the complex (β-CD-Chol/RES) for 0 (T0), 24, and 48 h. CTR is the vehicle DMSO control. β-CD-Chol alone was used at identical amounts as those in the corresponding β-CD-Chol/RES complex (indicated as v10 and v100, respectively). (**A**) Wound closure effects are normalized to the vehicle control (CTR, illustrated as a dashed blue line) and quantified as described in *Methods*. *, *p* < 0.05 and **, *p* < 0.01 vs. CTR; n.s., not significant. (**B**) Representative images at 4× magnifications.

To explore the biological utility of β-CD-Chol as a nanocarrier for RES, wound-healing assays were conducted employing human keratinocyte HaCaT cells (Figure 6A). As expected, RES (100 nM) significantly promoted wound closures over a 48 h period (Figure 6B). Treatments with RES (100 nM) delivered by β-CD-Chol mimicked the pro-migratory effects of RES, and after 48 h induced a significantly higher closure of HaCaT cell wounds compared to the vehicle control condition (Figure 6B). β-CD-Chol alone did not significantly alter HaCaT cell migration (Figure 6), suggesting that, at the amount employed in the present studies, β-CD-Chol is devoid of biological activities on human wound kinetics. Importantly, no significant differences in migratory effects were observed between RES and RES into the β-CD-Chol at any time point examined (Figure 6A), indicating that β-CD-Chol did not perturb the biological actions of RES.

## 3. Materials and Methods

### 3.1. Materials

All reagents were from commercial sources and were used without further purifications. Resveratrol (RES) and diphenyl picrylhydrazyl (DPPH) were purchased from Sigma-Aldrich (Milan, Italy).

### 3.2. Instrumentation

Centrifugation was carried out by the Heraeus Pico 21 centrifuge (Thermo Scientific, Thermo Fisher, Waltham, MA, USA). Samples were freeze-dried using a Lyoquest-85 (Telstar, Milan, Italy). UV–vis spectra were recorded on a UV–VIS spectrophotometer (8453 UV–Visible spectrophotometer; Agilent Technologies, Santa Clara, CA, USA) and a Jasco v770 UV vis/NIR spectrophotometer (JASCO Europe Srl, Cremella LC, Italy). NMR spectra of the complex (β-CD-Chol at 6 mM concentration) were acquired on a Bruker 400^TM^ spectrometer (Bruker, Ettlingen, Germany), and chemical shifts (*δ*) are reported in parts per million (ppm) using the residual solvent signal as the internal calibration standard. The 2D-ROESY NMR spectra were acquired at a 300 ms mixing time. LED UV chamber^TM^ UWAVE (Villebon-sur-Yvette, France) was used to irradiate the samples with a lamp centered at 365 nm and an irradiance of 10 mW/cm^2^. An HPLC VWR HITACHI Chromaster 5430 Diode Array Detector system (VWR International Srl, Milan, Italy) was used to detect and quantify *trans*-to-*cis* isomerization.

### 3.3. Synthesis of β-CD-Chol

The mono-choline-β-CD-Chol (β-CD-Chol) was synthesized as previously reported [28]. Mono-Tosyl-β-CD (60 mg, 0.046 mmol), placed in a test tube under argon atmosphere, was added, with *N*,*N*-dimethylethanolamine (850 μL) used as a reagent solvent. The reaction mixture was stirred at 80 °C in an inert environment overnight. The reaction mixture was dried and then purified on a reversed phase column (Lichroprep 40–63 μm RP-18) to eliminate the reaction by-products. Water (200 mL) was used as an eluent, and the fractions containing the desired product were pooled and dried under vacuum at 40 °C. The solid residue was dissolved in water, placed on an ion exchange resin column (Dowex 50), and eluted with 3% aqueous ammonium bicarbonate (300 mL). Salts were removed by thermal decomposition, carried out by repeated vacuum evaporation with water. The solid residue was dissolved in water and passed through an ion exchange resin column (Dowex 1) in chloride form. The pure product was obtained as a white powder (45% yield). The ^1^H NMR (400.13 MHz, D_2_O, 297 K) signals were consistent with the expected structure and data reported in reference [27].

### 3.4. Preparation of β-CD-Chol/RES Inclusion Complex

For the preparation of the inclusion complex, 2 mg of β-CD-Chol (1.6 µmol) was solubilized in 1 mL of phosphate-citrate buffer (pH 6) at room temperature, then an excess of resveratrol (1 mg, 4.4 µmol) was added. The mixture was sonicated for 30 min and then magnetically stirred for 3 days. Then, it was centrifugated at 10,000 rpm for 15 min to remove any non-solubilized resveratrol and the supernatant was recovered. The amount of solubilized RES was determined by UV–vis spectrophotometer (50 μL of sample + 750 μL H_2_O/EtOH 1:1 *v*/*v*) by the absorbance value at 306 nm, referring to an extinction molar coefficient of 26,000 M^−1^ cm^−1^. The complex can be lyophilized to obtain a solid-state complex.

### 3.5. Phase Solubility Study

Resveratrol (0.6 mg, 2.6 µmol) was added to six vials containing β-CD-Chol at different concentrations (from 0 to 2.0 × 10^−3^ M in citrate-phosphate buffer, pH 6). The samples were sonicated in a water bath for 15 min and magnetically stirred at 25 °C for 3 days. Subsequently, the dispersions were centrifuged at 10,000 rpm for 15 min in order to eliminate the non-solubilized RES. The supernatant of each sample was recovered and analyzed by a UV–vis spectrophotometer (50 μL of sample + 750 μL H_2_O/EtOH 1:1 *v*/*v*). The amount of RES was determined by the absorbance value at 306 nm. All measurements were performed in triplicate. The data obtained were used for determining the inclusion constant of the complex (*K_c_*) and the complexation efficiency (*CE*) by using the following equations:

(1) $$K_{c}=\frac{Slope}{S_{0}(1-Slope)}$$

(2) $$CE=\frac{Slope}{1-Slope}$$

### 3.6. Water Solubility and Dissolution Rate Determination

The degree of water solubility of the complex was determined by suspending the lyophilized complex (20 mg) in water (200 µL) and stirring a 25 °C for 24 h. The suspension was centrifugated (10,000 rpm, 15 min) and the supernatant was analyzed by UV–vis spectrophotometry (306 nm). The dissolution rate was assessed by dissolving the lyophilized β-CD-Chol/RES complex (2 mg/0.1 mg) and RES powder (0.1 mg, 0.4 µmol) in 1 mL of citrate-phosphate buffer (pH 6). The samples were placed in a shaker at 37 °C and shaken at 400 rpm. Aliquots of 100 μL were taken at 5, 10, 20, 30, 40, and 50 min and centrifuged to remove any undissolved drug. At each sampling time, a volume of buffer solution equal to that taken was added and the correction for cumulative dilution was calculated. The concentration of RES in the centrifuged solutions was determined by a UV–vis spectrophotometer at λ 306 nm. Each aliquot of sample (50 μL) was diluted with 750 μL of H_2_O/EtOH (1:1 *v*/*v*) in order to extract RES from the complex.

### 3.7. Molecular Modeling Simulations

Computational calculations were performed with the software xtb (extended Tight Binding, version 6.6.1) [47]. The structure of RES, β-CD-Chol, and the inclusion complexes β-CD-Chol/RES were optimized with the GFN2-xTB method to a very-tight level (--opt vtight) [48] and with implicit water (--alpb water) [49]. Two types of RES orientations were considered on the base of which of the two different aromatic rings, mono-hydroxylated (Mono-OH form) or di-hydroxylated (Di-OH form), is inside the cavity and pointing toward the narrow edge of the cyclodextrin. For each orientation, the conformation with the lowest energy has been taken into account. Analysis of interatomic distances was executed with Mercury (2024.3.1-build 428097-Copyright© CCDC, 2021–2024, Cambridge, UK).

### 3.8. Evaluation of DPPH Radical Scavenging Activity

The radical scavenging activities of RES and RES in β-CD-Chol were estimated by DPPH assay [50,51]. RES, ranging from 0 to 30.2 µM concentration, was reacted with 0.05 mM DPPH in 2 mL of methanol/aqueous citrate-phosphate buffer (1.8:0.2 *v*/*v*, respectively). Each solution was stirred at 25 °C and its absorbance at λ = 517 nm was monitored over a period of 2 h. Because changes in absorbance after 30 min were minimal, the percentage of radical scavenging activity (*RSA*%) was calculated using the values at that time by the following equation:

(3) $$RSA\%=\frac{A_{0}-A_{S}}{A_{0}}\times 100$$

where $A_{0}$ and $A_{S}$ are the DPPH absorbances in the absence and presence of pure RES or β-CD-Chol/ RES, respectively.

The amount of RES required to scavenge half of the initial DPPH concentration (SC_50_) was calculated by regression analysis of RSA % versus the RES concentration (R^2^ ≥ 0.996). Finally, the antiradical power, defined as the reciprocal of SC_50_ (ARP = 1/SC_50_), was also estimated. Each experiment was carried out in triplicate and data are expressed as mean ± standard deviation (SD). The results were analyzed by variance analysis (ANOVA) and the mean values were compared by Tukey’s test at a significance level of 0.05.

### 3.9. Photostability Study

1 mL of RES solution (0.1 mM) and β-CD-Chol/RES solution (RES 0.1 mM) were placed into a UV–LED chamber. The samples were irradiated with a lamp at λ 365 nm for 30 min or exposed to sunlight for 7 days. The amounts of *trans*-RES after irradiation were determined by HPLC. Chromatographic analysis was performed by injecting 20 μL of sample, appropriately diluted (50 μL sample + 750 μL of H_2_O/EtOH, 1:1 *v*/*v*), on a Kinetex chromatographic column (250 × 4.6 mm) with C18 stationary phase (5 μm, 100 Å) maintained at 20 °C. The elution conditions were as follows: isocratic, eluent 25:75 acetonitrile: H_2_O (TFA 0.1%), flow rate 1.0 mL/min. The detection of *trans*- and *cis*-RES was performed at 306 nm wavelength and the retention times were 13 min and 19 min for *trans*- and *cis*-RES, respectively. The percentage of *trans*-RES retained after irradiation was calculated by the following equation:

(4) $$trans\;RES=\frac{C_{t}}{C_{0}}\times 100$$

where *C*_0_ is the initial concentration of *trans*-resveratrol and *C_t_* is the concentration of *trans*-resveratrol after irradiation.

### 3.10. Cell Cultures

Human keratinocyte HaCaT cell lines were purchased from the commercial cell bank of Istituto Zooprofilattico Sperimentale di Lombardia ed Emilia Romagna (IZSLER, Brescia, Italy). Cells (passages 1–6) were maintained at 37 °C (5% CO_2_) in DMEM medium containing 10% fetal bovine serum (FBS), 2 mM of L-glutamine, 100 units/mL of penicillin, and 100 µg/mL of streptomycin. All media and reagents were from Euroclone S.p.A. (Pero, Milan, Italy).

### 3.11. Wound-Healing Assay

HaCaT cells (5 × 10^4^ cells/well) were seeded into 24-well plates and grown to confluence. Cell monolayers were then scratched with a pipet tip to create wounds of ~1 mm width. After washing (3 times) with PBS to remove cell debris, wounded monolayers were incubated for 48 h (37 °C in complete medium) in the presence of the indicated treatments. Time courses (0, 24, and 48 h) of wound closures were carried out by photography (at 4× magnifications) with a phase-contrast microscope and quantified with the MRI-Wound-Healing-Tools plugin of the NIH-Image-J (software version 1.51k) (ImageJ, National Institutes of Health, Bethesda, MD, USA). Data were calculated as % of wound closures with respect to the correspondent conditions at time 0 and expressed as the normalized values with respect to the control condition treated with the vehicle.

### 3.12. Statistical Analysis for Human Cell Studies

Results are shown as mean ± SEM of three independent experiments, performed in duplicate. Statistical comparisons were performed by two way-ANOVA, and *p* values were considered significant at α ≤ 0.05. All analyses were done with GraphPad Prism 10.4.1 (GraphPad Software, Inc., San Diego, CA, USA).

## 4. Conclusions

The present study describes the formation of an inclusion complex of resveratrol and a cationic β-cyclodextrin derivative functionalized with one choline moiety. The formation of the inclusion complex was carried out by a simple phase solubility method, without use of organic solvent. The formation of a 1:1 inclusion complex was demonstrated, and constant stability (K_C_ 2051 M^−1^) and complexation efficiency (0.23) were determined. The complexation markedly improved the water solubility of resveratrol. Molecular modeling simulations, according to cross-correlation peaks in 2D-ROESY NMR spectra, suggested two lowest-energy structures for the inclusion complex. The inclusion into the cyclodextrin improved the RES dissolution rate and reduced the photoinduced *trans*-to-*cis* isomerization, while preserving its radical scavenging and wound-healing activities. This work paves the way for further studies aiming to evaluate mono-choline β-cyclodextrin as a carrier for drug delivery, especially to cells overexpressing choline transporters.

## Data Availability

The datasets used and analyzed during the current study are available from the corresponding author, on reasonable request.
